# (*E*)-1-{4-[Bis(4-bromo­phen­yl)meth­yl]piperazin-1-yl}-3-(4-eth­oxy-3-meth­oxy­phen­yl)prop-2-en-1-one

**DOI:** 10.1107/S1600536811052123

**Published:** 2011-12-14

**Authors:** Yan Zhong, XiaoPing Zhang, Bin Wu

**Affiliations:** a School of Chemistry and Chemical Engineering, Southeast University, Sipailou No. 2 Nanjing, Nanjing 210096, People’s Republic of China; b Centre of Laboratory Animals, Nanjing medical University, Hanzhong Road No. 140 Nanjing, Nanjing 210029, People’s Republic of China; c School of Pharmacy, Nanjing Medical University, Hanzhong Road No. 140 Nanjing, Nanjing 210029, People’s Republic of China

## Abstract

In the title mol­ecule, C_29_H_30_Br_2_N_2_O_3_, the piperazine ring has a chair conformation and the C=C double bond has an *E* conformation. The dihedral angle between the bromo­benzene rings is 79.1 (3)°. In the crystal, mol­ecules are linked through C—H⋯O and C—H⋯Br hydrogen bonds.

## Related literature

For a related structure and background to cinnamic acid derivatives, see: Teng *et al.* (2011 ▶); Zhong *et al.* (2011 ▶). For further synthetic details, see: Wu *et al.* (2008 ▶). For puckering parameters, see: Cremer & Pople (1975 ▶).
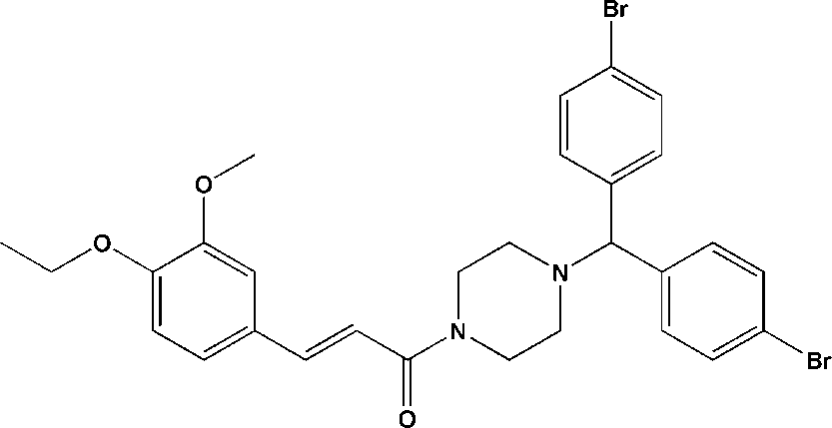


## Experimental

### 

#### Crystal data

C_29_H_30_Br_2_N_2_O_3_
*M* *_r_* = 614.37
Triclinic, 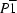

*a* = 8.5520 (17) Å
*b* = 10.355 (2) Å
*c* = 16.361 (3) Å
α = 92.85 (3)°
β = 100.52 (3)°
γ = 95.25 (3)°
*V* = 1415.3 (5) Å^3^
*Z* = 2
Mo *K*α radiation
μ = 2.90 mm^−1^
*T* = 293 K
0.20 × 0.10 × 0.10 mm

#### Data collection

Enraf–Nonius CAD-4 diffractometer
Absorption correction: ψ scan (North *et al.*, 1968 ▶) *T*_min_ = 0.595, *T*_max_ = 0.761
5569 measured reflections
5190 independent reflections
2233 reflections with *I* > 2σ(*I*)
*R*_int_ = 0.098
3 standard reflections every 200 reflections  intensity decay: 1%

#### Refinement

*R*[*F* ^2^ > 2σ(*F* ^2^)] = 0.077
*wR*(*F* ^2^) = 0.145
*S* = 1.01
5190 reflections
325 parameters
H-atom parameters constrained
Δρ_max_ = 0.28 e Å^−3^
Δρ_min_ = −0.29 e Å^−3^

### 

Data collection: *CAD-4 EXPRESS* (Enraf–Nonius, 1994 ▶); cell refinement: *CAD-4 EXPRESS*; data reduction: *XCAD4* (Harms & Wocadlo, 1995 ▶); program(s) used to solve structure: *SHELXS97* (Sheldrick, 2008 ▶); program(s) used to refine structure: *SHELXL97* (Sheldrick, 2008 ▶); molecular graphics: *SHELXTL* (Sheldrick, 2008 ▶); software used to prepare material for publication: *SHELXL97*.

## Supplementary Material

Crystal structure: contains datablock(s) I, global. DOI: 10.1107/S1600536811052123/su2344sup1.cif

Structure factors: contains datablock(s) I. DOI: 10.1107/S1600536811052123/su2344Isup2.hkl

Supplementary material file. DOI: 10.1107/S1600536811052123/su2344Isup3.cml

Additional supplementary materials:  crystallographic information; 3D view; checkCIF report

## Figures and Tables

**Table 1:** Hydrogen-bond geometry (Å, °)

*D*—H⋯*A*	*D*—H	H⋯*A*	*D*⋯*A*	*D*—H⋯*A*
C12—H12*A*⋯O1^i^	0.93	2.55	3.358 (8)	145
C20—H20*A*⋯O1^ii^	0.93	2.57	3.461 (8)	161
C16—H16*B*⋯Br1^iii^	0.97	2.79	3.562 (7)	137

Symmetry codes: (i) 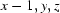; (ii) 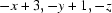; (iii) 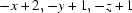.

**Fig. 1.**
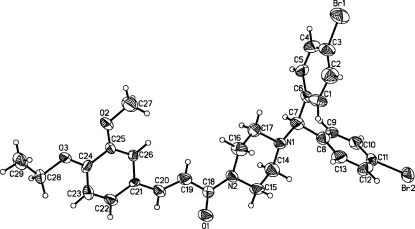
The molecular structure and numbering scheme of the title molecule. Displacement ellipsoids are drawn at the 50% probability level.

**Fig. 2.**
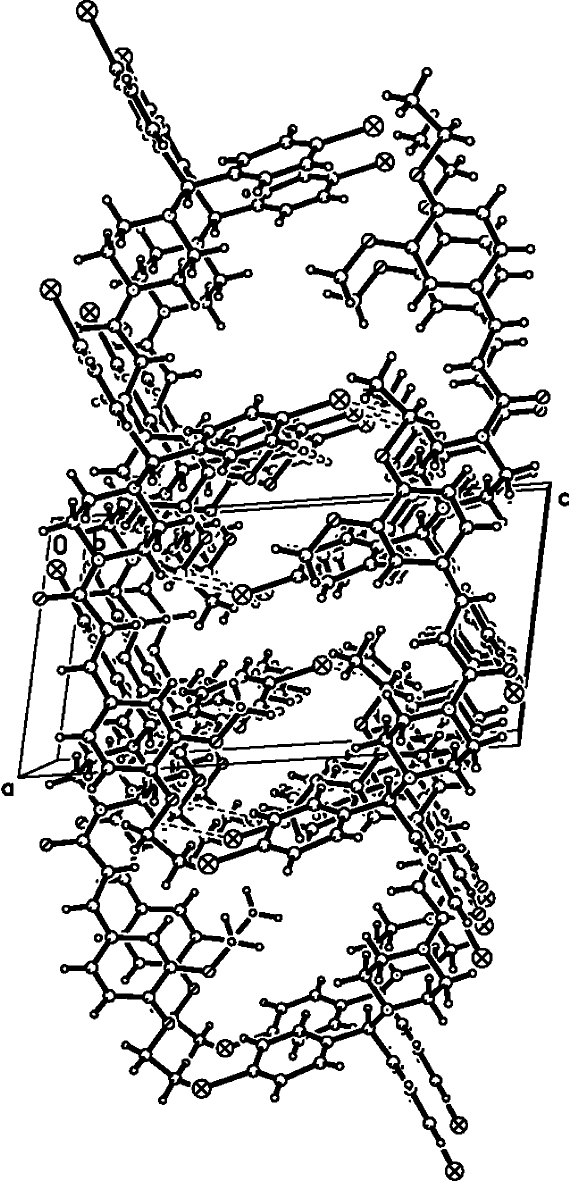
Crystal packing of the title compound viewed along the b axis. The C—H···O and C—H···Br hydrogen bonds are shown as dashed lines.

## supplementary crystallographic information

### Comment

As a continuation of our study of cinnamic acid derivatives (Teng *et al.*, 2011; Zhong *et al.*, 2011), we report herein on the synthesis and crystal structure of the title compound (Fig. 1). All the bond lengths and angles are normal and correspond to those observed in related compounds (Teng *et al.*, 2011; Zhong *et al.*, 2011). The molecule exists in an E configulation with respect to the C19═C20 ethene bond [1.321 (7) Å]. The piperazine ring adopts a chair conformation with puckering parameters (Cremer & Pople, 1975) Q = 0.543 (6) Å, θ = 5.5 (6) °, φ = 329 (7) °.

In the crystal, molecules are linked by intermolecular C—H···O and C—H···Br hydrogen bonds (Fig. 2 and Table 1).

### Experimental

The synthesis follows the method of Wu *et al.* (2008). The title compound was prepared by stirring a mixture of (*E*)-3-(4-ethoxy-3-metoxyphenyl)acrylic acid (0.889 g; 4 mmol), dimethyl sulfoxide (2 ml) and dichloromethane (30 ml) for 6 h at room temperature. The solvent was removed under reduced pressure. The residue was dissolved in acetone (15 ml) and reacted with 1-(bis(4-bromophenyl)methyl)piperazine (2.461 g; 6 mmol) in the presence of triethylamine (5 ml) for 12 h at room temperature. The resultant mixture was cooled. The title compound thus obtained was filtered and was recrystallized from ethanol. The colourless single crystals of the title compound used in the *x*-ray diffraction studies were grown in ethanol by slow evaporation at room temperature.

### Refinement

The C-bound H-atoms were included in calculated positions and treated as riding atoms: C-H = 0.93, 0.96, 0.97 and 0.98 Å for CH(aromatic), CH_3_, CH_2_ and CH(methine) H-atoms, respectively, with U_iso_(H) = k × U_eq_(parent C-atom), where k = 1.5 for CH_3_ H-atoms and k = 1.2 for all other H-atoms.

### Crystal data

**Table:**

C_29_H_30_Br_2_N_2_O_3_	*Z* = 2
*M**_r_* = 614.37	*F*(000) = 624
Triclinic, *P*1	*D*_x_ = 1.442 Mg m^−^^3^
Hall symbol: -P 1	Mo *K*α radiation, λ = 0.71073 Å
*a* = 8.5520 (17) Å	Cell parameters from 25 reflections
*b* = 10.355 (2) Å	θ = 9–13°
*c* = 16.361 (3) Å	µ = 2.90 mm^−^^1^
α = 92.85 (3)°	*T* = 293 K
β = 100.52 (3)°	Block, colourless
γ = 95.25 (3)°	0.20 × 0.10 × 0.10 mm
*V* = 1415.3 (5) Å^3^	

### Data collection

**Table:**

Enraf–Nonius CAD-4 diffractometer	2233 reflections with *I* > 2σ(*I*)
Radiation source: fine-focus sealed tube	*R*_int_ = 0.098
graphite	θ_max_ = 25.4°, θ_min_ = 1.3°
ω/2θ scans	*h* = 0→10
Absorption correction: ψ scan (North *et al.*, 1968)	*k* = −12→12
*T*_min_ = 0.595, *T*_max_ = 0.761	*l* = −19→19
5569 measured reflections	3 standard reflections every 200 reflections
5190 independent reflections	intensity decay: 1%

### Refinement

**Table:**

Refinement on *F*^2^	Primary atom site location: structure-invariant direct methods
Least-squares matrix: full	Secondary atom site location: difference Fourier map
*R*[*F*^2^ > 2σ(*F*^2^)] = 0.077	Hydrogen site location: inferred from neighbouring sites
*wR*(*F*^2^) = 0.145	H-atom parameters constrained
*S* = 1.01	*w* = 1/[σ^2^(*F*_o_^2^) + (0.045*P*)^2^] where *P* = (*F*_o_^2^ + 2*F*_c_^2^)/3
5190 reflections	(Δ/σ)_max_ < 0.001
325 parameters	Δρ_max_ = 0.28 e Å^−^^3^
0 restraints	Δρ_min_ = −0.29 e Å^−^^3^

### Special details

**Table:**

Geometry. All e.s.d.'s (except the e.s.d. in the dihedral angle between two l.s. planes) are estimated using the full covariance matrix. The cell e.s.d.'s are taken into account individually in the estimation of e.s.d.'s in distances, angles and torsion angles; correlations between e.s.d.'s in cell parameters are only used when they are defined by crystal symmetry. An approximate (isotropic) treatment of cell e.s.d.'s is used for estimating e.s.d.'s involving l.s. planes.
Refinement. Refinement of *F*^2^ against ALL reflections. The weighted *R*-factor *wR* and goodness of fit *S* are based on *F*^2^, conventional *R*-factors *R* are based on *F*, with *F* set to zero for negative *F*^2^. The threshold expression of *F*^2^ > σ(*F*^2^) is used only for calculating *R*-factors(gt) *etc*. and is not relevant to the choice of reflections for refinement. *R*-factors based on *F*^2^ are statistically about twice as large as those based on *F*, and *R*- factors based on ALL data will be even larger.

### Fractional atomic coordinates and isotropic or equivalent isotropic displacement parameters (Å^2^)

**Table:**

	*x*	*y*	*z*	*U*_iso_*/*U*_eq_	
Br1	0.65376 (13)	0.34766 (12)	0.57637 (6)	0.1418 (5)	
N1	0.9260 (5)	0.3258 (4)	0.2058 (3)	0.0541 (12)	
O1	1.3079 (5)	0.4867 (4)	0.0380 (3)	0.0802 (14)	
C1	0.7349 (9)	0.3675 (6)	0.3323 (4)	0.083 (2)	
H1A	0.7259	0.4241	0.2897	0.099*	
Br2	0.20032 (9)	−0.00602 (9)	0.02290 (5)	0.0987 (4)	
N2	1.1790 (6)	0.4652 (5)	0.1444 (3)	0.0610 (14)	
O2	1.9023 (5)	0.8508 (5)	0.3993 (3)	0.0900 (16)	
C2	0.6782 (10)	0.3959 (8)	0.4101 (5)	0.107 (3)	
H2A	0.6223	0.4676	0.4162	0.128*	
O3	2.1174 (5)	0.9315 (4)	0.3219 (2)	0.0730 (13)	
C3	0.7089 (10)	0.3160 (9)	0.4728 (4)	0.092 (3)	
C4	0.7929 (9)	0.2026 (9)	0.4636 (4)	0.102 (3)	
H4A	0.8159	0.1495	0.5076	0.123*	
C5	0.8378 (7)	0.1751 (7)	0.3896 (4)	0.075 (2)	
H5A	0.8928	0.1034	0.3820	0.091*	
C6	0.7996 (6)	0.2565 (5)	0.3260 (4)	0.0529 (15)	
C7	0.8396 (8)	0.2183 (7)	0.2417 (4)	0.080 (2)	
H7A	0.9100	0.1486	0.2499	0.096*	
C8	0.6895 (7)	0.1635 (6)	0.1820 (4)	0.0630 (17)	
C9	0.6436 (7)	0.0372 (6)	0.1727 (4)	0.0648 (17)	
H9A	0.7130	−0.0176	0.2001	0.078*	
C10	0.5035 (7)	−0.0188 (5)	0.1265 (4)	0.0641 (18)	
H10A	0.4772	−0.1083	0.1227	0.077*	
C11	0.4077 (6)	0.0579 (6)	0.0878 (3)	0.0526 (14)	
C12	0.4392 (8)	0.2000 (6)	0.0887 (4)	0.083 (2)	
H12A	0.3687	0.2529	0.0601	0.100*	
C13	0.5895 (8)	0.2469 (6)	0.1383 (4)	0.079 (2)	
H13A	0.6225	0.3354	0.1419	0.095*	
C14	0.9581 (8)	0.2932 (7)	0.1231 (4)	0.083 (2)	
H14A	1.0273	0.2237	0.1263	0.100*	
H14B	0.8585	0.2622	0.0859	0.100*	
C15	1.0330 (8)	0.4036 (6)	0.0898 (4)	0.083 (2)	
H15A	0.9571	0.4677	0.0793	0.099*	
H15B	1.0592	0.3760	0.0368	0.099*	
C16	1.1500 (8)	0.4937 (6)	0.2289 (4)	0.085 (2)	
H16A	1.0802	0.5625	0.2282	0.103*	
H16B	1.2504	0.5235	0.2658	0.103*	
C17	1.0756 (7)	0.3766 (6)	0.2603 (4)	0.0732 (19)	
H17A	1.1491	0.3102	0.2647	0.088*	
H17B	1.0553	0.3979	0.3156	0.088*	
C18	1.3040 (7)	0.5159 (7)	0.1128 (4)	0.0684 (18)	
C19	1.4415 (8)	0.5873 (6)	0.1696 (4)	0.074 (2)	
H19A	1.4322	0.6116	0.2238	0.089*	
C20	1.5779 (7)	0.6173 (6)	0.1446 (4)	0.074 (2)	
H20A	1.5825	0.5853	0.0911	0.089*	
C21	1.7193 (6)	0.6923 (5)	0.1888 (3)	0.0443 (13)	
C22	1.8438 (7)	0.7364 (6)	0.1511 (4)	0.0641 (17)	
H22A	1.8393	0.7109	0.0953	0.077*	
C23	1.9733 (7)	0.8157 (7)	0.1922 (4)	0.079 (2)	
H23A	2.0502	0.8475	0.1627	0.094*	
C24	1.9934 (6)	0.8501 (6)	0.2765 (4)	0.0588 (16)	
C25	1.8700 (6)	0.8058 (6)	0.3175 (4)	0.0539 (15)	
C26	1.7401 (6)	0.7287 (5)	0.2764 (3)	0.0533 (15)	
H26A	1.6621	0.6984	0.3056	0.064*	
C27	1.7825 (8)	0.8123 (7)	0.4451 (4)	0.096 (2)	
H27A	1.8132	0.8494	0.5014	0.144*	
H27B	1.7701	0.7192	0.4453	0.144*	
H27C	1.6831	0.8420	0.4198	0.144*	
C28	2.2484 (7)	0.9702 (7)	0.2786 (4)	0.078 (2)	
H28A	2.2110	1.0169	0.2301	0.094*	
H28B	2.2975	0.8951	0.2614	0.094*	
C29	2.3669 (8)	1.0595 (7)	0.3454 (4)	0.104 (3)	
H29A	2.4591	1.0890	0.3230	0.156*	
H29B	2.3993	1.0119	0.3935	0.156*	
H29C	2.3156	1.1331	0.3612	0.156*	

### Atomic displacement parameters (Å^2^)

**Table:**

	*U*^11^	*U*^22^	*U*^33^	*U*^12^	*U*^13^	*U*^23^
Br1	0.1442 (9)	0.2104 (13)	0.0742 (6)	−0.0019 (8)	0.0470 (6)	−0.0167 (7)
N1	0.058 (3)	0.061 (3)	0.040 (2)	−0.009 (2)	0.007 (2)	−0.001 (2)
O1	0.090 (3)	0.084 (3)	0.061 (3)	−0.029 (3)	0.017 (3)	0.014 (3)
C1	0.135 (7)	0.050 (4)	0.071 (5)	0.003 (4)	0.038 (5)	0.020 (4)
Br2	0.0657 (5)	0.1419 (8)	0.0789 (5)	−0.0321 (5)	0.0134 (4)	−0.0091 (5)
N2	0.059 (3)	0.071 (3)	0.049 (3)	−0.019 (3)	0.016 (3)	0.000 (3)
O2	0.068 (3)	0.134 (4)	0.069 (3)	−0.025 (3)	0.026 (3)	0.028 (3)
C2	0.120 (7)	0.109 (7)	0.090 (6)	0.003 (6)	0.022 (6)	−0.006 (6)
O3	0.059 (3)	0.107 (4)	0.051 (2)	−0.017 (2)	0.013 (2)	0.026 (2)
C3	0.106 (6)	0.113 (7)	0.046 (4)	−0.034 (5)	0.018 (4)	−0.017 (5)
C4	0.106 (7)	0.148 (9)	0.046 (4)	−0.003 (6)	0.000 (4)	0.005 (5)
C5	0.065 (4)	0.094 (5)	0.064 (4)	0.004 (4)	−0.001 (4)	0.033 (4)
C6	0.057 (3)	0.035 (3)	0.065 (4)	−0.018 (3)	0.015 (3)	0.016 (3)
C7	0.074 (5)	0.097 (6)	0.066 (4)	−0.017 (4)	0.012 (4)	0.020 (4)
C8	0.067 (4)	0.065 (4)	0.058 (4)	0.009 (3)	0.014 (3)	0.001 (3)
C9	0.067 (4)	0.074 (4)	0.058 (4)	0.015 (3)	0.012 (3)	0.030 (4)
C10	0.075 (4)	0.044 (4)	0.081 (5)	0.014 (3)	0.024 (4)	0.031 (3)
C11	0.048 (3)	0.058 (3)	0.056 (4)	0.007 (3)	0.022 (3)	−0.004 (3)
C12	0.090 (5)	0.080 (5)	0.080 (5)	0.027 (4)	0.001 (4)	0.034 (4)
C13	0.094 (5)	0.063 (4)	0.075 (5)	−0.020 (4)	0.009 (4)	0.028 (4)
C14	0.085 (5)	0.091 (6)	0.071 (5)	−0.011 (4)	0.019 (4)	−0.004 (4)
C15	0.083 (5)	0.098 (5)	0.057 (4)	−0.034 (4)	0.002 (4)	0.032 (4)
C16	0.106 (6)	0.073 (5)	0.069 (5)	−0.031 (4)	0.015 (4)	0.003 (4)
C17	0.066 (4)	0.088 (5)	0.058 (4)	−0.012 (4)	0.005 (3)	−0.008 (4)
C18	0.050 (4)	0.100 (5)	0.060 (4)	0.015 (3)	0.013 (3)	0.022 (4)
C19	0.083 (5)	0.073 (4)	0.064 (4)	−0.018 (4)	0.012 (4)	0.026 (4)
C20	0.065 (4)	0.096 (5)	0.061 (4)	−0.019 (4)	0.020 (4)	0.017 (4)
C21	0.044 (3)	0.057 (3)	0.039 (3)	0.029 (3)	0.007 (3)	0.029 (3)
C22	0.074 (4)	0.067 (4)	0.062 (4)	0.016 (3)	0.029 (3)	0.028 (3)
C23	0.046 (4)	0.103 (6)	0.081 (5)	−0.022 (4)	0.013 (4)	0.008 (4)
C24	0.039 (3)	0.056 (4)	0.074 (4)	−0.004 (3)	−0.011 (3)	0.029 (3)
C25	0.039 (3)	0.070 (4)	0.056 (4)	0.003 (3)	0.018 (3)	0.019 (3)
C26	0.040 (3)	0.059 (3)	0.065 (4)	0.002 (2)	0.010 (3)	0.037 (3)
C27	0.099 (6)	0.108 (6)	0.086 (5)	0.005 (5)	0.034 (5)	0.009 (5)
C28	0.046 (3)	0.103 (5)	0.086 (5)	0.000 (4)	0.015 (4)	0.024 (4)
C29	0.088 (5)	0.114 (6)	0.101 (6)	−0.045 (5)	0.028 (5)	−0.012 (5)

### Geometric parameters (Å, °)

**Table:**

Br1—C3	1.862 (7)	C13—H13A	0.9300
N1—C17	1.458 (6)	C14—C15	1.438 (7)
N1—C14	1.458 (7)	C14—H14A	0.9700
N1—C7	1.484 (7)	C14—H14B	0.9700
O1—C18	1.254 (7)	C15—H15A	0.9700
C1—C6	1.328 (8)	C15—H15B	0.9700
C1—C2	1.469 (9)	C16—C17	1.475 (7)
C1—H1A	0.9300	C16—H16A	0.9700
Br2—C11	1.934 (5)	C16—H16B	0.9700
N2—C18	1.346 (7)	C17—H17A	0.9700
N2—C15	1.468 (7)	C17—H17B	0.9700
N2—C16	1.469 (7)	C18—C19	1.471 (8)
O2—C25	1.365 (6)	C19—C20	1.321 (7)
O2—C27	1.416 (7)	C19—H19A	0.9300
C2—C3	1.354 (10)	C20—C21	1.429 (7)
C2—H2A	0.9300	C20—H20A	0.9300
O3—C24	1.369 (6)	C21—C22	1.379 (7)
O3—C28	1.467 (6)	C21—C26	1.438 (7)
C3—C4	1.447 (11)	C22—C23	1.364 (8)
C4—C5	1.360 (9)	C22—H22A	0.9300
C4—H4A	0.9300	C23—C24	1.383 (8)
C5—C6	1.384 (7)	C23—H23A	0.9300
C5—H5A	0.9300	C24—C25	1.406 (7)
C6—C7	1.524 (8)	C25—C26	1.355 (7)
C7—C8	1.509 (8)	C26—H26A	0.9300
C7—H7A	0.9800	C27—H27A	0.9600
C8—C9	1.324 (7)	C27—H27B	0.9600
C8—C13	1.400 (8)	C27—H27C	0.9600
C9—C10	1.358 (8)	C28—C29	1.550 (8)
C9—H9A	0.9300	C28—H28A	0.9700
C10—C11	1.297 (7)	C28—H28B	0.9700
C10—H10A	0.9300	C29—H29A	0.9600
C11—C12	1.470 (8)	C29—H29B	0.9600
C12—C13	1.419 (8)	C29—H29C	0.9600
C12—H12A	0.9300		
			
C17—N1—C14	108.8 (5)	N2—C15—H15B	108.8
C17—N1—C7	112.4 (5)	H15A—C15—H15B	107.6
C14—N1—C7	114.4 (5)	N2—C16—C17	110.5 (5)
C6—C1—C2	116.4 (7)	N2—C16—H16A	109.5
C6—C1—H1A	121.8	C17—C16—H16A	109.5
C2—C1—H1A	121.8	N2—C16—H16B	109.5
C18—N2—C15	121.1 (5)	C17—C16—H16B	109.5
C18—N2—C16	126.0 (5)	H16A—C16—H16B	108.1
C15—N2—C16	110.8 (5)	N1—C17—C16	111.7 (5)
C25—O2—C27	114.8 (5)	N1—C17—H17A	109.3
C3—C2—C1	118.8 (8)	C16—C17—H17A	109.3
C3—C2—H2A	120.6	N1—C17—H17B	109.3
C1—C2—H2A	120.6	C16—C17—H17B	109.3
C24—O3—C28	115.8 (5)	H17A—C17—H17B	107.9
C2—C3—C4	121.2 (7)	O1—C18—N2	118.5 (6)
C2—C3—Br1	122.7 (8)	O1—C18—C19	121.9 (6)
C4—C3—Br1	116.1 (6)	N2—C18—C19	119.0 (6)
C5—C4—C3	118.8 (7)	C20—C19—C18	121.0 (6)
C5—C4—H4A	120.6	C20—C19—H19A	119.5
C3—C4—H4A	120.6	C18—C19—H19A	119.5
C4—C5—C6	118.3 (7)	C19—C20—C21	128.7 (6)
C4—C5—H5A	120.8	C19—C20—H20A	115.6
C6—C5—H5A	120.8	C21—C20—H20A	115.6
C1—C6—C5	125.9 (7)	C22—C21—C20	123.0 (5)
C1—C6—C7	116.6 (6)	C22—C21—C26	115.3 (5)
C5—C6—C7	117.5 (6)	C20—C21—C26	121.7 (5)
N1—C7—C8	111.1 (5)	C23—C22—C21	122.8 (6)
N1—C7—C6	113.5 (5)	C23—C22—H22A	118.6
C8—C7—C6	109.9 (5)	C21—C22—H22A	118.6
N1—C7—H7A	107.4	C22—C23—C24	121.7 (6)
C8—C7—H7A	107.4	C22—C23—H23A	119.1
C6—C7—H7A	107.4	C24—C23—H23A	119.1
C9—C8—C13	118.1 (6)	O3—C24—C23	125.5 (5)
C9—C8—C7	121.6 (6)	O3—C24—C25	117.2 (6)
C13—C8—C7	120.3 (6)	C23—C24—C25	117.2 (5)
C8—C9—C10	125.3 (6)	C26—C25—O2	128.0 (5)
C8—C9—H9A	117.4	C26—C25—C24	121.0 (6)
C10—C9—H9A	117.4	O2—C25—C24	111.0 (5)
C11—C10—C9	117.1 (6)	C25—C26—C21	121.9 (5)
C11—C10—H10A	121.4	C25—C26—H26A	119.0
C9—C10—H10A	121.4	C21—C26—H26A	119.0
C10—C11—C12	125.6 (6)	O2—C27—H27A	109.5
C10—C11—Br2	122.2 (5)	O2—C27—H27B	109.5
C12—C11—Br2	112.2 (4)	H27A—C27—H27B	109.5
C13—C12—C11	112.1 (5)	O2—C27—H27C	109.5
C13—C12—H12A	124.0	H27A—C27—H27C	109.5
C11—C12—H12A	124.0	H27B—C27—H27C	109.5
C8—C13—C12	121.8 (6)	O3—C28—C29	103.1 (5)
C8—C13—H13A	119.1	O3—C28—H28A	111.1
C12—C13—H13A	119.1	C29—C28—H28A	111.1
C15—C14—N1	111.6 (6)	O3—C28—H28B	111.1
C15—C14—H14A	109.3	C29—C28—H28B	111.1
N1—C14—H14A	109.3	H28A—C28—H28B	109.1
C15—C14—H14B	109.3	C28—C29—H29A	109.5
N1—C14—H14B	109.3	C28—C29—H29B	109.5
H14A—C14—H14B	108.0	H29A—C29—H29B	109.5
C14—C15—N2	114.0 (5)	C28—C29—H29C	109.5
C14—C15—H15A	108.8	H29A—C29—H29C	109.5
N2—C15—H15A	108.8	H29B—C29—H29C	109.5
C14—C15—H15B	108.8		
			
C6—C1—C2—C3	6.4 (11)	C18—N2—C15—C14	144.2 (6)
C1—C2—C3—C4	−1.2 (12)	C16—N2—C15—C14	−51.3 (8)
C1—C2—C3—Br1	176.3 (5)	C18—N2—C16—C17	−144.8 (6)
C2—C3—C4—C5	−1.7 (12)	C15—N2—C16—C17	51.7 (8)
Br1—C3—C4—C5	−179.4 (5)	C14—N1—C17—C16	59.4 (7)
C3—C4—C5—C6	−0.5 (10)	C7—N1—C17—C16	−172.9 (6)
C2—C1—C6—C5	−9.3 (10)	N2—C16—C17—N1	−57.7 (8)
C2—C1—C6—C7	172.6 (6)	C15—N2—C18—O1	−14.6 (10)
C4—C5—C6—C1	6.5 (10)	C16—N2—C18—O1	−176.5 (6)
C4—C5—C6—C7	−175.5 (6)	C15—N2—C18—C19	174.4 (6)
C17—N1—C7—C8	−177.2 (5)	C16—N2—C18—C19	12.4 (10)
C14—N1—C7—C8	−52.5 (8)	O1—C18—C19—C20	−3.6 (10)
C17—N1—C7—C6	58.3 (7)	N2—C18—C19—C20	167.1 (6)
C14—N1—C7—C6	−177.0 (5)	C18—C19—C20—C21	175.7 (6)
C1—C6—C7—N1	47.2 (8)	C19—C20—C21—C22	−168.1 (7)
C5—C6—C7—N1	−130.9 (6)	C19—C20—C21—C26	11.0 (10)
C1—C6—C7—C8	−77.8 (7)	C20—C21—C22—C23	175.1 (6)
C5—C6—C7—C8	104.0 (7)	C26—C21—C22—C23	−4.0 (8)
N1—C7—C8—C9	140.8 (6)	C21—C22—C23—C24	4.5 (10)
C6—C7—C8—C9	−92.7 (8)	C28—O3—C24—C23	−8.4 (9)
N1—C7—C8—C13	−43.0 (9)	C28—O3—C24—C25	176.7 (5)
C6—C7—C8—C13	83.4 (7)	C22—C23—C24—O3	−178.3 (5)
C13—C8—C9—C10	−2.7 (10)	C22—C23—C24—C25	−3.4 (10)
C7—C8—C9—C10	173.6 (6)	C27—O2—C25—C26	−0.9 (9)
C8—C9—C10—C11	0.6 (10)	C27—O2—C25—C24	179.1 (5)
C9—C10—C11—C12	1.0 (9)	O3—C24—C25—C26	177.7 (5)
C9—C10—C11—Br2	−177.6 (4)	C23—C24—C25—C26	2.3 (9)
C10—C11—C12—C13	−0.4 (9)	O3—C24—C25—O2	−2.3 (8)
Br2—C11—C12—C13	178.3 (5)	C23—C24—C25—O2	−177.6 (6)
C9—C8—C13—C12	3.2 (10)	O2—C25—C26—C21	177.8 (5)
C7—C8—C13—C12	−173.1 (6)	C24—C25—C26—C21	−2.2 (8)
C11—C12—C13—C8	−1.7 (9)	C22—C21—C26—C25	2.9 (7)
C17—N1—C14—C15	−57.0 (7)	C20—C21—C26—C25	−176.3 (5)
C7—N1—C14—C15	176.4 (5)	C24—O3—C28—C29	−179.9 (5)
N1—C14—C15—N2	54.4 (8)		

### Hydrogen-bond geometry (Å, °)

**Table:**

*D*—H···*A*	*D*—H	H···*A*	*D*···*A*	*D*—H···*A*
C12—H12A···O1^i^	0.93	2.55	3.358 (8)	145
C20—H20A···O1^ii^	0.93	2.57	3.461 (8)	161
C16—H16B···Br1^iii^	0.97	2.79	3.562 (7)	137

Symmetry codes: (i) *x*−1, *y*, *z*; (ii) −*x*+3, −*y*+1, −*z*; (iii) −*x*+2, −*y*+1, −*z*+1.
